# Lifestyles associated with malaria in pregnancy in northwest Colombia: a mixed study from Latin American critical epidemiology

**DOI:** 10.1186/s12936-024-05046-2

**Published:** 2024-07-24

**Authors:** Jaiberth Antonio Cardona-Arias, Luis Felipe Higuita-Gutiérrez, Jaime Carmona-Fonseca

**Affiliations:** 1 School of Microbiology, University of Antioquia Medellín, Medellín, Colombia; 2 School of Medicine, School of Microbiology, Universidad Cooperativa de Colombia, Universidad de Antioquia, Medellín, Colombia; 3 School of Medicine, Universidad de Antioquia Research Group “Salud y Comunidad-César Uribe Piedrahita”, Universidad de Antioquia, Medellín, Colombia

**Keywords:** Malaria, Pregnancy, Critical epidemiology, Social determination of health, Mixed study, Psychometrics, Ethnography

## Abstract

**Background:**

In the scientific literature on Malaria in Pregnancy (MiP), no studies have been conducted on lifestyles based on critical theory. The objective of this study was to analyse the lifestyles or singular processes of social determination of health in MiP in northwestern Colombia.

**Methods:**

Mixed QUAN-QUAL convergent triangulation study. In the quantitative component, a psychometric evaluation and a cross-sectional design were conducted in 400 pregnant women to whom the Pender-Walker lifestyle scale and a survey on MiP prevention were applied. In the qualitative study, a critical ethnography was conducted with 46 pregnant women in whom their narratives and practices regarding lifestyles at home and healthcare were described.

**Results:**

The frequency of MiP was 9%, and a higher occurrence of the disease was identified in those who did not control stagnant water (29%), did not use insecticide-treated net (16%) and went to the hospital (14%) or the microscopist (20%) when they had fever. This coincides with the presence of unhealthy lifestyles, little knowledge about malaria, and a low perception of the risk of getting sick, as well as meanings and experiences about MiP, maternity, and pregnancy that show a high clinical, cultural, and socioeconomic burden for the women studied.

**Conclusion:**

This epidemiological profile and the approach to lifestyles based on the postulates of critical theory in health evidence that pregnant women exposed to malaria suffer serious social, cultural and health injustices that are not possible to impact with the current health model of malaria control in Colombia guided by aetiopathogenic, biomedical, positivist and utilitarian theories.

## Background

Critical epidemiology, as part of critical theory in health, is defined as a group of ideas and practices cohesive by materialist, historical, and cultural interests that reveal the effects of economic relations of accumulation and power in capitalism on health-disease profiles. The central categories are society-nature metabolism, social reproduction, and social determination of health (SDH) [1–4].

The SDH tends toward an explanation and understanding of epidemiological profiles under the following premises: (i) healthdisease is a materialist dialectical process, where the singular corresponds to personal or family lifestyles, the particular refers to the ways of life of classes and social groups, and the general refers to the mode of production of a society; (ii) individual and group specificities are determined by the social, cultural, political, and historical context (subsumption of the simple into the complex, and of the biological into the social); (iii) to confront the social order that is reproduced from the general to the singular, individuals and groups produce movements of tension and struggle (relative autonomy) against the *status quo* [1–4].

The singular level of SDH is the most immediate in the process of discovering reality. At this level, the frequency and distribution of health-disease problems are identified, and lifestyles are studied as a central construct. In SDH, lifestyles are defined as the biological, psychological, and social components of the person and the family, the conceptions-meanings about personal values, and the management of actions for the protection of health [1, 5].

Since the beginning of Latin American critical epidemiology in the 1970s, the main authors of the SDH approach have focused on their theoretical, epistemological, and political positioning, with incipient research development. For example, in Colombia until the beginning of 2023, only thirteen investigations had been conducted from this perspective of public health, most of them qualitative studies, and none on Malaria in Pregnancy (MiP) [6–18]. Only one study has been published in the world applying the SDH approach to investigate MiP [19]. In Colombia, the available systematic reviews show that research on MiP focuses on the vector, parasite, or immune response, with few epidemiological studies, without mixed studies, and no research on lifestyles associated with MiP [20, 21].

MiP increases the risk of maternal anemia, severe malaria, and death. MiP also affects fetal health by causing intrauterine growth retardation and abortion. In newborns, MiP can cause congenital malaria, premature birth, low birth weight, anemia, malnutrition, increased risk of other infections, and death [22–24]. Furthermore, in 2020, there were 248 million pregnancies in the world, of which 157 million (63%) were registered in 85 malaria-endemic countries and 122 million (49%) occurred in areas with transmission of the parasite; in this last group, there were an estimated 1.4 million (1%) stillbirths, 33.5 million (27%) induced abortions, and 16.1 million (13%) spontaneous abortions [25]. These epidemiological consequences of MiP present high heterogeneity highlighting the level of endemicity, mother's immunity, social factors, and various lifestyles associated with the use of preventive methods and health services [22, 26, 27].

Investigating lifestyle factors associated with MiP (or singular processes of SDH) is relevant because it allows the design of specific interventions for its prevention and control, aimed at modifying or reinforcing the attitudes and behaviors of the individuals, families, or social groups facing MiP. This becomes more important when considering that in Colombia the health system has several actors with different responsibilities to guarantee health care: (i) Ministry of Health, through the administrator of the resources of the general system of social security in health, transfers the money to the Health Promoter Entity (HPE) and to the municipalities to pay for the healthcare, (ii) HPE is responsible for the affiliation of people to the social security in health, organizing and guaranteeing the provision of health services by payment to Health Services Provider Institutions (HSPI) (hospitals, laboratories and clinics that provide the care service to patients), (iii) HSPI through contracting or charging the municipality or HPE, guarantee the provision of diagnostic, control and treatment services for diseases such as MiP to the entire population that demands health services, mainly through a passive system: focused in symptomatic people who go to the HSPI for care or treatment. HPE and HSPI have the responsibility of intervening lifestyles of their patients as part of their programs of disease prevention and health promotion, through different actions to mitigate risky lifestyles and promote healthy lifestyles [19].

Therefore, the objective of this study was to analyse the lifestyles or processes related to the singular level of SDH associated with MiP in northwestern Colombia.

## Methods

### Study type

Mixed QUAN + QUAL convergent triangulation, in which the quantitative and qualitative components are designed and implemented in parallel or independently, and the articulation occurs in the interpretation of the results [28]. The QUAN component was developed using psychometric evaluation and a cross-sectional epidemiological design. The QUAL component consisted of a particularist critical ethnographythat focuses on investigating dominant discourses in vulnerable populations to propose their transformation when these intensify social inequalities of power or sustain unjust socioeconomic, political, and cultural configurations (that do not adjust to human rights or to the social justice) [29, 30].

### Study place

**Fig. 1:**
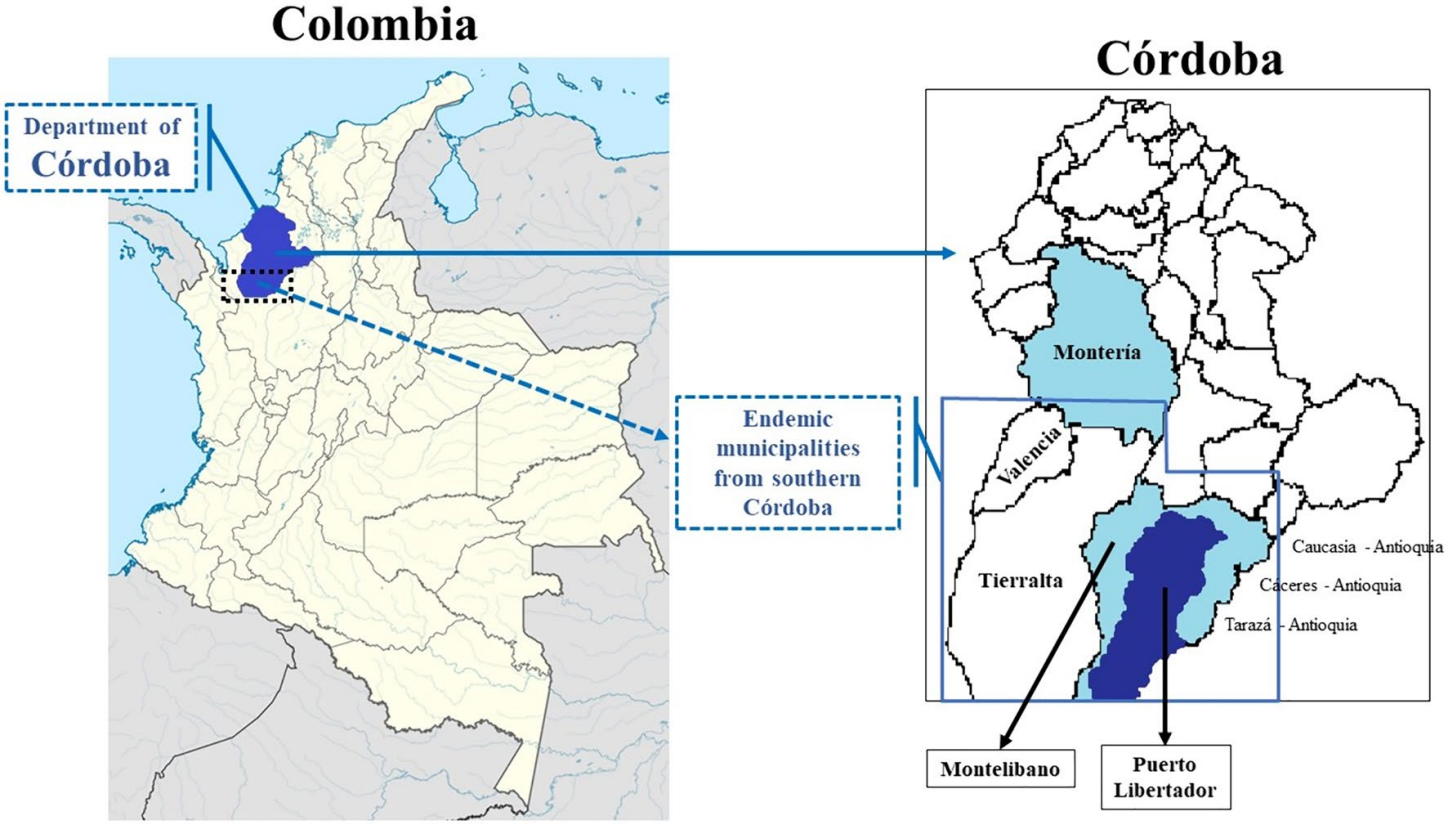
Site of study

Malaria in Colombia is concentrated in the Pacific region (departments of Chocó, Cauca and Nariño) with 50–60% of cases, the northwestern zone in the departments of Antioquia (Urabá and Bajo Cauca) and Córdoba with 20–30%, and the remaining percentage corresponds to cases from the Amazon (5–10%) and Orinoquía—Central East and Atlantic [31]. In the department of Córdoba, nearly 7000 cases of malaria are registered per year, which are concentrated in the southern municipalities of Valencia, Tierralta, Montelíbano and Puerto Libertador; in the latter, this study was developed in which 924 cases of malaria occurred in 2017 and 1950 during 2018 [32]; without specifying the proportion of pregnant women affected (Fig. 1).

### Subjects of the study

The cross-sectional research was conducted with 400 pregnant women; these correspond to the total number of women who attended the antenatal control programme during 2022 (no sample size or sampling was applied because it corresponds to the total population of pregnant women in the hospital). The coverage of the antenatal control program in the municipality is greater than 90%; 100% is not achieved because some pregnant women choose to perform the antenatal control in Montería (capital of the department) or in other municipalities that are closer to their places of residence in rural areas (Montelíbano in the same department, or Caucasia, Cáceres and Tarazá in the department of Antioquia) (Fig. 1), or they are indigenous people who prefer care through traditional medicine, or women who make portability in their health care (care of some health services such as gynecology-obstetrics in municipalities other than that of residence).

The critical ethnography was developed with 46 pregnant women between January 2022 and March 2023, including pregnant women not infected with malaria and positive for MiP. The number was established by theoretical saturation (addressing the multidimensionality of lifestyles), a priori*,* and inductive themes (addressing the properties and dimensions of pre-established and emergent categories), when the new interviews repeated aspects of previous participants [33]. To guarantee selection for maximum variation in the design, pregnant women from different health affiliation regimes were included with a wide age range (14–36 years), educational level (primary and secondary), and area of residence (urban or rural).

In both QUAN-QUAL components, the eligibility criteria were as follows: permanent residence in the municipality, attendance at the antenatal control programme at the local hospital, not having a diagnosis of gynecological or obstetric diseases or complications that would prevent them from answering the survey or attending the interview, acceptance to voluntarily participate in the study, and signing informed consent or assent.

### Information collection

In the QUAN component, lifestyle was studied using Pender-Walker construct, which has been validated for Colombian pregnant women [34]. The Pender-Walker Health Promotion Model is widely used because it allows us to comprehend human behaviors related to health and promote healthy behaviours. This construct consists of 6 dimensions: responsibility for health (with 7 items), physical activity (with 8 items), nutrition (with 8 items), spiritual growth (with 9 items), interpersonal relationships (with 9 items), and stress management (with 7 items). In addition, some lifestyle factors associated with MiP were investigated through a survey designed and validated by the group of researchers. The clinical chart of the pregnant women was reviewed to identify whether a thick blood smear was performed in their antenatal control, and the results were extracted.

In the QUAL component, semi-structured interviews were conducted to investigate meanings and experiences related to pregnancy, lifestyles at home, and health care, through general questions: (i) tell me what your pregnancy process has been like, (ii) what you do on a normal day; (iii) tell me about the malaria and antenatal control programmes. This was complemented with participant observations during the interviews to describe the interaction with the interviewer and aspects that could affect the interview (personal traits of the pregnant woman such as shyness or mistrust, interference from health workers or family members, among others). Non-participant observation was also conducted in hospital waiting rooms, rooms designated in hospitals for health education, and local health posts to analyze the interactions between pregnant women and health workers and their communication mechanisms. All of the above was complemented by a field diary completed by one researcher (JACA) with reflective, methodological, and theoretical notes.

### Rigor criteria

In the QUAN component, selection biases were controlled with the application of eligibility criteria, information biases with the application of a survey with face validity, the Pender-Walker construct with evaluation of reproducibility and psychometric validity, and a thick blood smear was performed in local hospitals by qualified personnel, following the instructions of the National Institute of Health of Colombia.

In the QUAL component, the guidelines of holism (capturing the variations of the categories), contextualization (describing the region of study), reflexivity (contrasting and discussing interpretations and theoretical frame predominant in each researcher), articulation of the -emic (describes the facts from the point of view of its agents) and -etic (describes the facts from the point of view of the observer) perspectives, saturation of categories, and triangulation of methods, researchers, and theories were used. In addition, credibility criteria were followed through prolonged contact with participants, auditability of researchers' interpretations and validation of results with participants, and transferability or applicability to other endemic areas [29, 30, 33].

### Information analysis

The variables were described with frequencies (# and %) and summary measures (median and ranges). The psychometric evaluation of the lifestyle construct was performed with reliability (Cronbach's alpha), internal consistency (Spearman’s Rho Correlation between each item and the dimension of the lifestyle construct to which it belongs), discriminant power (Spearman's Rho Correlation between each item with the dimensions of lifestyle to which it does not belong), construct validity with factorial loadings (λ coefficients), and proportion of the explained variance obtained in an exploratory factor model. The frequency of MiP according to the categorical variables was compared using the Chi-square test, and the strength of association was estimated using prevalence ratios (and their confidence intervals). The comparison of MiP with continuous variables was performed using the Mann–Whitney U test because non-compliance assumption of normality (according to Kolmogorov–Smirnov with correction of Lilliefors). With the variables that presented statistically significant differences, a multivariate adjustment model was used to control for confounding variables; this was done with a generalized log-binomial family model. The statistical analyses were performed using SPSS 29.0.

In the QUAL component, all the material was transcribed to encode and categorize using pre-established and emerging codes, and the content of each category was analysed through its properties (attributes that delimit the content of each category) and dimensions (range of variation of a property). This phenomenological-hermeneutical insight concluded with an explanation of semantic networks between categories. As a synthesis, QUAL data were triangulated with QUAN to specify the main lifestyles (or singular processes of the SDH) associated with MiP.

### Ethical considerations

The ethical principles of the Declaration of Helsinki and Resolution 8430 of Colombia were applied. This study was classified as minimal risk and was approved by the Ethics Committee of the SIU (*in Spanish Sede de Investigación Univesitaria*), University of Antioquia, Minute 21-101-961. The participants signed informed consent (of legal age) or assent (under 18 years of age), which was obtained in writing; it was also signed by a witness (external to the research group) and a member of the health team who explained its content. When it was possible, parental consent was obtained; however, according to rulings C-246/17 and T-675-17 on the self-determination of minors, the Constitutional Court of the Republic of Colombia in 2017 defined that parental consent is not necessary in these cases given that at 14 years of age, it has been established that minors may have the maturity to begin assuming obligations and responsibilities in society, such as marriage, consenting to sexual relations, and the right to privacy in the family environment.

## Results

### QUAN (Quantitative) component

The median age was 22 years with an interquartile range of 18–28 and range 14–44 years. The median number of years of education was 10, with an interquartile range 7–11.

In the group studied, 44% (n = 177) of the pregnant women had malaria at some moment in their lives, 61% (n = 243) had a thick blood smear test performed during their current pregnancy, and among them, the frequency of MiP was 9.1% (n = 22) (95%CI 5.2–12.9). In group, 97% (n = 387) were affiliated with the subsidized regime, half (n = 202) were residents of an urban area, and the main occupation was homemaker (n = 373. In practices related to malaria in general, 79% (n = 318) self-medicate when experiencing fever, only 26% (n = 103) go to the hospital when experiencing this symptom, and 62% (n = 250) avoid the presence of mosquitoes in their home. In villages (rural area) and neighborhoods (urban area), there are few practices to prevent malaria. Regarding specific knowledge about MiP, the lowest proportions were found in the following items: MiP can be cured with plants (45%, n = 180) and it is caused by swimming in rivers and ponds (50%, n = 199) (Table 1).

**Table 1:** Socio-health description, knowledge, and practices about MiP in pregnant women studied

Variables and their categories		n	%
Malaria diagnosis	You have had malaria in your life	177	44.3
	You have made a thick blood smear during this pregnancy	243	60.8
	You have been diagnosed with malaria during her current pregnancy	22	9.1^b^
Origin	Rural	198	49.5
	Urban	202	50.5
Health affiliation	Subsidized regime (financed by the state)	387	96.8
	Contributory regime (financed by company and worker)	13	3.3
Occupation of the pregnant woman	Homemaker	373	93.3^a^
	Student	22	5.5^a^
	Various trades	12	3.0
	Professional	7	1.8
Practices related to malaria	Self-medicate when you have fever	318	79.5
	Go to the hospital if you have a fever	103	25.8
	Go to the microscopist if you have a fever	90	22.5
	Avoiding stagnant water in your home	352	88.0
	Prevent the presence of mosquitoes in your home	250	62.5
	Use insecticide-treated net to sleep	121	30.3
	Use of mosquito repellent	93	23.3
	The municipality or hospital has performed fumigation actions in the community	30	7.5
	Their neighbourhood or village engages in activities to prevent malaria	27	6.8
	Their neighbourhood or village takes action to diagnose malaria	13	3.3
Knowledge about malaria in pregnancy	MiP is a preventable	353	88.3
	MiP can be cured with plants	180	45.0
	MiP is a serious-severe problem	392	98.0
	MiP can affect the fetus	354	88.5
	MiP can cause death	388	97.0
	MiP is caused by swimming in rivers and ponds	199	49.8
	MiP is caused by NOT using insecticide-treated nets or repellents	258	64.5
	MiP is caused by changes in the climate	240	60.0
	The main symptoms of MiP are fever and chills	391	97.8
	The main symptoms of MiP are headache and myalgia	357	89.3

^a^3.5% (n = 14) are householders and students

^b^It corresponds to 5.5% of the total studied and 9.1% of those in whom a thick blood smear was applied in the current pregnancy

The Pender-Walker lifestyle construct presented excellent reliability, internal consistency, discriminant power, and construct validity, with some items that reduced the overall value of the statistical parameters of the psychometric properties: (i) in the nutrition dimension, it was the consumption of fats and cholesterol (Rho = 0.30 in discriminant power) and eating breakfast, lunch, and dinner every day (λ = 0.213 in construct validity); (ii) in stress management it was the use of specific methods to manage tension (Rho = 0.27 and λ = 0.22) and practice relaxation or meditation 15–20 min daily (Rho = 0.31 and λ = 0.27), and (iii) in growth spiritual, the item indicated feeling united with a greater force (for example God) (Rho = 0.24) (Table 2).

**Table 2:** Psychometric properties of the healthy lifestyles scale and description of the scores of the six dimensions of the Pender-Walker scale

Dimension	Reliability α Cronbach	Internal consistency Rho^a^	Discriminating power Rho^b^	Explained variance %	Construct validity λ^c^	Score Me (IR)^d^
Physical activity	0.92	0.69–0.82	0.01–0.36	65.1	0.48–0.90	0 (0–13)
Nutrition	0.67	0.30–0.77	0.01–0.32	40.5	0.21–0.65	56 (52–67)
Stress management	0.66	0.27–0.69	0.02–0.27	41.0	0.22–0.74	54 (42–71)
Relationships	0.80	0.41–0.68	0.01–0.31	50.6	0.45–0.64	54 (38–67)
Health responsibility	0.80	0.49–0.67	0.07–0.37	50.9	0.52–0.70	58 (46–75)
Spiritual growth	0.82	0.24–0.72	0.03–0.30	53.7	0.37–0.67	92 (79–100)

^a^Range of Spearman’s Rho correlations item-dimension to which it belongs

^b^Range of Spearman’s Rho correlations item-dimension to which it does NOT belong

^c^Range of coefficient λ or factor loadings of the items of each dimension

^d^Median and interquartile range (0 indicates the worst result and 100 the top result)

In the lifestyle profile, the lowest score was found in the physical activity dimension (the majority do not perform physical activities), the highest score was recorded in the spiritual growth dimension, and the remaining four dimensions recorded regular scores (Table 2). In addition, pregnant women reported working (mainly in household activities) every day of the week (range 5–7 days). The median number of hours they worked in a day was 5 with an interquartile range of 4–6 and range 2- 11. On a typical day, the median number of hours of rest was 4 with an interquartile range of 3–5 and a range of 1–8; this time was dedicated to watching television and sleeping.

The frequency of MiP did not present statistical differences with the following variables: health affiliation regime (*p* Chi^2^ = 0.788), self-medication in cases of fever (*p* Chi^2^ = 0.781), use of repellents (*p* Chi^2^ = 0.264) or other measures to avoid mosquitoes in the neighborhood or home (*p* Chi^2^ = 0.731), or knowledge about MiP (*p* Chi^2^ > 0.05), except for considering that MiP is preventable (*p* Chi^2^ = 0.045).

The frequency of MiP showed statistical differences depending on several factors, being higher in the groups that recorded the following aspects: going to the hospital (2.0 times) and to the microscopist when they have fever (3.8 times), not using insecticide-treated net (2.9 times), and not applying actions to avoid the presence of stagnant water (4.2 times). Based on the dimensions of lifestyles, statistical differences were only found in the dimensions of health responsibility and stress management, which were better among the positive women (Table 3). After performing the multivariate adjustment, only the use of insecticide-treated net (Prevalence ratio = 3.00 95%CI 1.02–8.4) and avoiding stagnant water retained their statistical significance (Prevalence ratio = 11.68 95%CI 3.07–44,64).

**Table 3:** Socio-health characteristics and lifestyles in pregnant women with and without malaria

		Prevalence MiP % (n)	Prevalence ratio (95% IC)	*p* Chi^2^
Characteristics				
Go to the hospital if you have a fever	Yes	14.0 (8)	2.0 (1.0–4.6)*	0.049*
	No	7.0 (13)		
Go to the microscopist if you have a fever	Yes	20.4 (11)	3.8 (1.7–8.6)**	< 0.001**
	No	5.3 (10)		
Use insecticide-treated net to sleep	No	15.6 (12)	2.9 (1.3–6.5)**	0.009**
	Yes	5.4 (9)		
Avoid stagnant water in your home	No	28.6 (6)	4.2 (1.8–9.7)**	0.001**
	Yes	6.8 (15)		

	With MiP	Without MiP	*p* M-W^b^
	Me (IR)^a^	Me (IR)^a^	
Lifestyles			
Physical activity	0 (0–0)	0 (0–17)	0.290
Nutrition	63 (56–70)	56 (52–67)	0.254
Stress management	71 (46–79)	52 (42–63)	0.014*
Relationships	58 (42–71)	50 (38–63)	0.208
Health responsibility	71 (54–83)	58 (50–75)	0.017*
Spiritual growth	92 (88–100)	92 (79–100)	0.139

^*^p < 0.05. ^**^p < 0.01

^a^Me (IR): Median and interquartile range

^b^Mann–Whitney U Test

### QUAL (Qualitative) component

The critical ethnography ennabled to comprehend the main meanings and experiences related to motherhood, health care, and MiP. In the **meanings of motherhood**, all participants referred to positive aspects, and only negative meanings related to two situations were found. First, among pregnant women who live in rural areas and have low monetary incomes, despite enthusiastically welcoming a new life, meanings associated with fear about the possibility of survival and the quality of life of the child predominate, due to the precarious material conditions of life. Second, in pregnant adolescents, negative meanings emerged related to doubts about the parenting process because the youngest women considered that without the support of their mothers, they would not know how to protect their children.

In the positive meanings of motherhood, three aspects were highlighted: (i) motherhood materializes the most important role of women in their families and communities, which is the reproduction of the family; (ii) it strengthens the life project and personal identity (dignity), in which it is essential to be a mother because the social and cultural construction of Being a Woman revolves around the exclusivity that women have for the conception of a new life. Some pregnant women indicated that they did not have a life plan before pregnancy, but after knowing that they would be mothers, this changes a little because they are pressured to think about their new role as mothers, since their family group and the community expects that the woman's daily life revolves around caring for the child; (iii) it allows to enhance some aspects of the emotional life of women and their husband, or to correct deficiencies in this dimension, because the child increases the emotional balance.

*“When I found out about the pregnancy, because I am a single mother, it is always scary, but I always have to accept it because a baby cannot be rejected, nor do anything against him/her, nor mistreat it; you always have to move forward. But he/she is always greeted with concern about the economic situation in which one is in [poverty]”* (24 year-old wife, she lives in a rural area)*.*

*“When I knew I was pregnant, I felt scared. I'm still scared because I don’t know how to be a mother. But when the baby moves he/she is very cute, and all worries are forgotten. But I don't know, I feel happy because it is something that all women want: to be a mother at some point in our lives. But I also stay scared because I don't know what to do when the child is sick, or because I don't know how to care for him/her. Or financially, not being able to give my son everything he needs”* (17 year-old homemaker and student, lives in a rural area)*.*

*“Motherhood is one of the most important stages of a woman. The most wonderful thing that women can have is the power to have children. Because that is one's main family. For me, it is the most beautiful stage of life”* (23 year-old pregnant woman, she works as a secretary and lives in an urban area)*.*

In combination with the above, the meanings of pregnancy presented various properties and dimensions, but the most common were to refer to it as another stage in a woman's life, a normal fact that is expected to be a woman, especially if she has a stable partner in whom the use of contraceptives is disregarded because it is expected that the woman can materialize her “*mission*” of becoming a mother and reproducing the family. For these reasons, in some pregnant women (mainly the youngest and those with monetary poverty), pregnancy does not generate important changes in the behaviour and care of pregnant women, and simultaneously, it is indicated that pregnancy is a process that, in most cases, is lived in loneliness, as an exclusive charge to women, because men are absent or limited to the material provision of the home. This situation had a few exceptions in three women with a high level of education and in whom the father was more present; although in these cases, it was also indicated that, due to cultural issues in the region, it is common that the man does not participate in antenatal care because this “*is a women's issue*”.

*“I didn’t have any changes in my life or any special care. During pregnancy, I maintained my normal life. I didn’t change anything, the food, the stillness, or anything else. I went to the nutritionist once because the doctor told me, that it is, the routine, go to the nutritionist, to the psychologist, or those appointments, but for the rest of my life, I continued my normal life. I only went to the check-up for the exams and nothing else”* (Pregnant woman with training as a technologist, lives in an urban area)*.*

*“I knew when I was two months pregnant, and I started the check-ups at 6 months. And I usually come alone. I came with my sister to the first checkups, but she no longer accompanies me, and I come alone”* (Pregnant woman with technical training, lives in an urban area)*.*

*“My spouse is present. Well, he is vigilant about the obligations that he has, and that's all. He is not very expressive, he does not touch my belly. He does nothing, just the economical thing, and that's all. He is not very affectionate, it is economically vigilant, and that's it”* (Pregnant woman with technical training, lives in an urban area)*.*

The above is related to the last category referring to the meanings of maternal and child health in which traditional care (family and community) is decisive; in this category, the following findings were highlighted: (i) preference for traditional medicine in indigenous pregnant women, and in those who reside in dispersed rural areas, with geographic and economic barriers to attending the hospital, (ii) coexistence of care based on the biomedical actions of the antenatal care, and other traditional ones from the home or rural communities, and (iii) from the perspective of pregnant women, antenatal care is focused on the care of the baby; therefore, the care of the pregnant woman is highly dependent on the advice of the families and traditions of their community (especially in remote areas) with actions based on home herbal remedies.

When investigating the meanings of malaria, its causes, transmission, prevention, and treatment, very poor or reductionist results were found, most pregnant women restrict these domains to the following sequence of events or knowledge: malaria is a disease caused by “the mosquito”, its prevention is based on vector control (for which only three strategies were cited: avoiding stagnant water, fumigating and using insecticide-treated net), and in cases where the infection could not be avoided, it is enough to go to station diagnosis or to the hospital to have a thick blood smear and obtain treatment. When attempting to delve deeper into each of these aspects, no new narratives or complementary information were found, which allowed us to conclude that the level of knowledge about malaria is poor in this population and is restricted to very basic biomedical information.

Specifically in MiP, the only issue that was different from that found for malaria in general was the risk of affecting the fetus, but not because the women conceived an intrauterine transmission of the parasite, but because of the link between the general state of health of the mother and that of the fetus. That is to say, MiP affects the mother, which creates a risk for the fetus due to how debilitating malaria can be for the pregnant woman, but not due to the direct effect of *Plasmodium* on the baby (the risk to fetal health is not directly caused by infection but is attributable to the mother's weakness).

*“I don’t know anything about malaria. Health workers did not tell me during my antenatal checkup that I should have something done for malaria. Maybe I know that if I get a fever, a lot of pain, and a lot of chills, I come here to the diagnostic station and pay $10,000 pesos for a thick blood smear, and if it comes back positive, I go to the hospital pharmacy. Otherwise, I don't hear anything about malaria in this town, nor is there almost anything about malaria prevention or care in my neighborhood”* (28 year-old wife, she lives in a rural area)*.*

*“Malaria is serious but not always. The truth is that we hardly talk about it here. In antenatal checkups, they have not talked to me about malaria, nor at school, I don't know anything about malaria, but I have never got sick from it”* (18-year-old wife, lives in an urban area).

*“Untreated malaria can kill the baby, because the mother becomes very decompensated and if she is unwell, the baby can die because it does not receive its nutrients from the mother”* (Secretary with technical training, age 22, lives in an urban area).

In combination with the above, the participants had a low perception of the risk of contracting MiP, because they limited their perception to the presence of fever and the abundance of mosquitoes. This influenced them to think that malaria is always symptomatic and is derived from seasons or places where large quantities of the vector are present (concepts of endemic or epidemic are not recognized, although malaria is endemic in the study area, with epidemic cycles). Testimonies illustrate the content of this category:

*“In my environment, in my home, in my work, there is nothing that endangers my life or my health. In general, everything is very healthy here, you don't see ugly or stagnant water, or flies, or anything like that”* (20 year-old wife, she lives in an urban area)*.*

*“On the issue of malaria, I don't have any risk, I don't see any risk in my environment. In malaria, a mosquito is required, so in the house it is enough to cover and wash the water tanks, so as not to encourage the mosquito, not to encourage the mosquito to come to me and infect me and my entire family. For rural people it is enough to use their awning, especially because in rural areas they are more vulnerable to acquiring this infection from water and other things that attract more mosquitoes”* (24 year-old wife, lives in an urban area)*.*

This situation is exacerbated by the finding that the diagnostic network and epidemiological surveillance do not function adequately and that in antenatal care, it is not common to include thick blood smears in quarterly control examinations (as indicated in the regulations for care of pregnant women), except those affiliated to the contributory regime who reported that during pregnancy they undergo at least two thick blood smear screenings. However, the most common thing is that the search for cases is only carried out in the presence of obvious symptoms such as intermittent fever or anaemia (doctors, most of them in their rural year, do not suspect this disease, which is why many cases are detected in advanced states), or on personal or family initiative, pregnant women go to the diagnosis station and pays to have the thick blood smear performed.

*“At home, I would get a fever, headache, and urinate red. From there, my spouse took me to the hospital. They performed a urine test, and the doctor gave me drugs for a urinary infection, but it wasn't that infection. Since I wasn't getting better, my spouse took me to another municipality, and there they tested me for malaria, and it came back positive. Because the malaria had already advanced, they sent me to Montería, and in Montería they put me in the intensive care unit for 10 days. I remember that they performed many exams and ultrasounds to ensure that the baby was okay. Then they had to donate blood to me because my platelets went low”* (20 year-old wife, she lives in a rural area)*.*

*“After being hospitalized for malaria, they told me I had to hang the awning. Then my spouse got the awning, and he bought it for like $30,000 pesos. I sleep with an awning every night. Before hospitalization, we did not use it because we did not think about it [malaria]”* (25 year-old wife, lives in an urban area).

*“I went to the antenatal checkups since I determined about the pregnancy. Since I am in the contributory regime, and my attention is in the private health institution, they gave me all the exams there. The bacteriologists gave me a thick blood smear at every checkup”* (28-year-old technologist, lives in an urban area)*.*

*“To diagnose malaria, you go to the village diagnostic station, and there they charge you $15,000 pesos for the thick blood smear, and then you seek treatment at the hospital or buy it on the sidewalk. It cost me $25,000 pesos. If you continue to have malaria, a week later you should take another thick blood smear to determine why you continue to have malaria. There I have to move again, and that costs $5,000 pesos by motorcycle and $8,000 by bus”* (18-year-old wife, lives in a rural area).

What has been stated in the previous paragraphs demonstrates a sequence of extremely worrying events that are reflected in the singular level of the SDH: (i) despite the high endemicity, the perception of the risk of infection is poor, (ii) lack of knowledge about the main factors of risk and consequences of MiP, (iii) diagnosis and treatment of MiP is a matter of person responsibility, implying high costs for families, (iv) absence of diagnostic actions for MiP, although the regulations indicate that antenatal care must include performing at least one thick blood smear every quarter, and *(v)* MiP care restricted to cases of high obstetric risk, symptomatic, or in advanced stages. These situations are complemented by the high use of traditional actions for fever management (MiP diagnosis is only resorted to when these fail).

*“For malaria, people drink herbal drinks. Because malaria rarely progresses, people use bitter drinks, and whoever can get their quinine drink. If that does not reduce the fever, they will look for someone who takes the thick blood smear”* (26 year-old wife, she lives in a rural area).

**Fig. 2:**
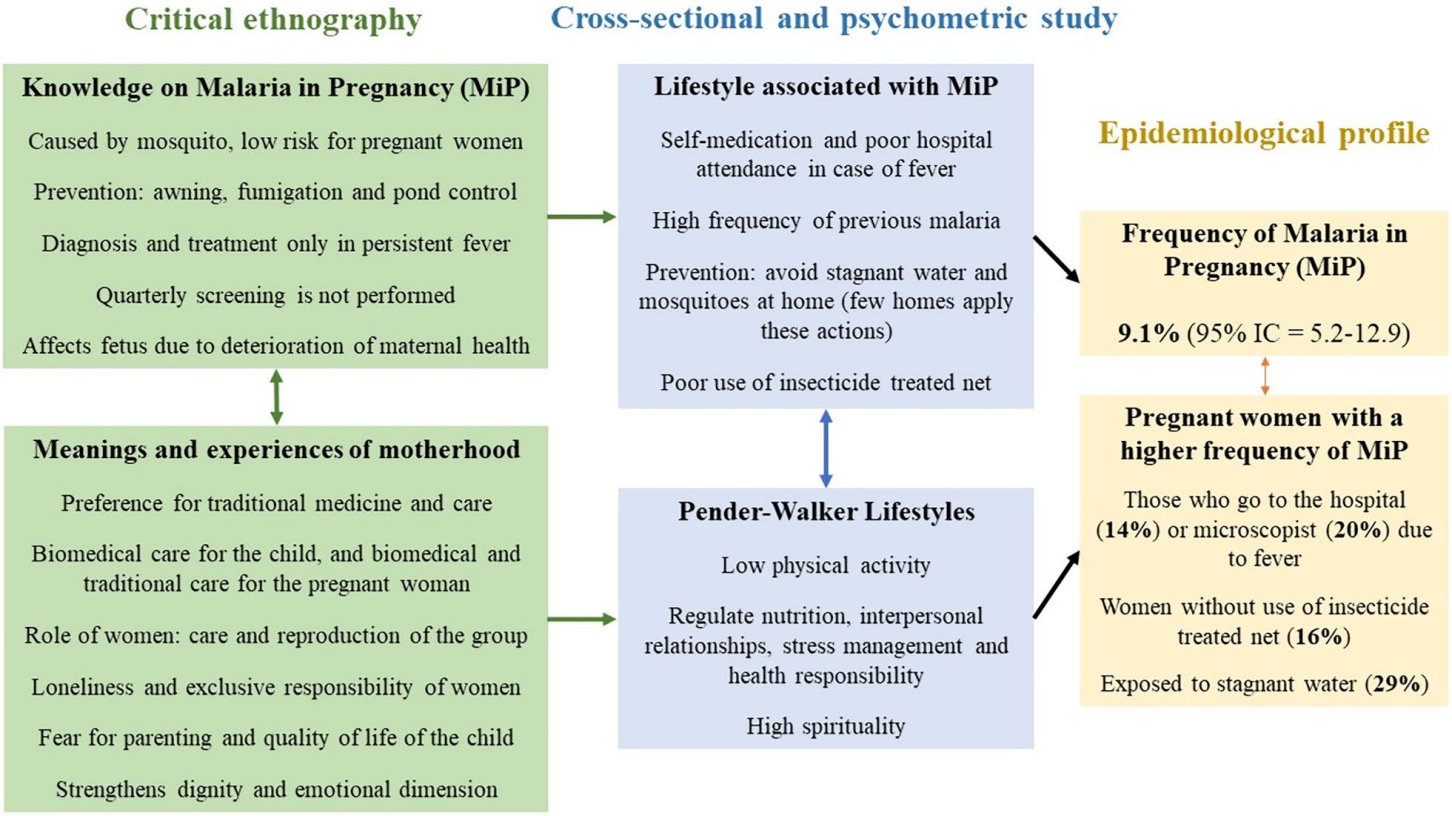
Integration of psychometric, epidemiological and ethnographic results

The articulation of the main ethnographic and epidemiological results is summarized in Fig. 2, in which three groupings are highlighted: (i) the main categories of the critical ethnography (differentiated with green) in relation to popular knowledge about MiP and the meanings of motherhood and pregnancy, (ii) the main data of the epidemiological study (blue) on lifestyles according to the Pender-Walker construct and those specific to MiP, (iii) the main findings from the epidemiological profile (yellow).

The frequency of MiP was 9%, and it was higher in pregnant women who visited the hospital (14%) or the microscopist (20%) when they had fever, in those who did not use ITNs (16%), and in those exposed to stagnant water (29%). This epidemiological profile was associated with unhealthy lifestyles linked to MiP, such as a high frequency of self-medication, low hospital attendance and ITN use, high exposure to malaria, and few actions to prevent mosquitoes. These results align with the presence of unhealthy lifestyles according to the Pender-Walker construct, with low scores in physical activity, nutrition, interpersonal relationships, stress management, and health responsibility.

These quantitative results are explained in greater depth by the qualitative findings, as the lifestyles associated with MiP are determined by beliefs about MiP, where the historical-cultural heritage is more important than the causal factors indicated by conventional epidemiology (QUAN component). This heritage related to MiP is characterized by the following findings:

i) The aetiological explanation focuses on the mosquitoes as the central and sufficient cause of MiP; consequently, preventive measures are based on vector control (ITN, fumigation, control of stagnant water), which is a responsibility of health authorities: the Ministry of Health, through the HPE, HSPI, and municipal health secretariats.
ii) Diagnosis and treatment of MiP are sought only in cases of persistent fever. Additionally, there is a lack of active case findings and quarterly screening in ANC. This highlights deficiencies in education, information, and health communication, limitations in preventive actions and epidemiological surveillance with community involvement, structural failures in malaria control programs and ANC that do not detect asymptomatic or mildly symptomatic cases. This increases the risk of clinical outcomes of MiP and leads to persistent parasite transmission.
iii) There is a lack of information about the risks to fetal and neonatal health from MiP. Pregnant women predominantly have more holistic and generic views about the good health of the fetus, which are linked to the mother’s overall health status.

These findings were expanded and deepened with the meanings and experiences of pregnancy and motherhood, revealing aspects that transcend the scope of the empiricist-functional approach of conventional epidemiology but are crucial for explaining and intervening in the epidemiological profile found and the consequences of MiP. It is clear that the epidemiological profile of MiP and the lifestyles associated with MiP and Pender-Walker (QUAN component) cannot be explained by interindividual variations or measures centered on individual responsibility, as has been the hegemonic discourse on healthy lifestyles. As indicated by the qualitative evidence of this study, in the studied area, structural problems that exceed the individual and their decisions persist and reproduce.

The qualitative findings demonstrate the social reproduction of knowledge, beliefs, meanings, and experiences that should be included in health policies as they determine the epidemiological profile. Additionally, these processes are not impacted by the contagionist-individualist theories that are hegemonic in MiP control. Among the sociocultural aspects found to account for this social reproduction are the following: infrastructure problems in the communities that generate stagnant water and mosquitoes, low coverage of biomedical services, few vector control actions and public policies, explanatory models of maternal–fetal health and MiP showing a preference for traditional medicine, an epidemiological profile of MiP determined by the sociocultural roles of pregnant women, and the primacy of concerns about the economic situation and quality of life of the child over MiP.

## Discussion

The frequency of MiP was 9.1%, which is higher than that obtained in a metanalysis on MiP that grouped data from 7932 pregnant women evaluated with thick blood smears in which a frequency of 5.8% was found [21]. This difference can be attributed to the endemic and epidemic heterogeneity of the Colombian territory and to the fact that the current study was conducted in one of the areas with the highest number of cases in the country. Furthermore, the meta-analysis grouped data from research contexts, including asymptomatic pregnant women, whereas the current study, in accordance with the findings of the QUAL component, had a low screening with thick blood smear, which is almost limited to symptomatic and advanced cases.

In the multivariate adjustment, the subgroups with a higher frequency of MiP were women who did not use the insecticide-treated net and those exposed to stagnant water, in whom the frequency increased to almost 30%. This is consistent with greater exposure to the vector and shows the relevance of the MiP control actions of the World Health Organization, which focus on prevention through the use of insecticide-treated nets, indoor residual spraying, and case search; these are effective but do not provide adequate coverage in endemic Colombian populations [35].

The epidemiological results of morbidity converge with a high frequency of risk lifestyles for MiP, such as self-medication, poor hospital attendance, and few preventive practices (mainly vector control) in pregnant women, which have been documented in other studies [36, 37]. These QUAN findings coincided and deepened in the QUAL component.

From the ethnographic perspective, the participants highlighted the absence of prevention and control actions in their places of residence (home and neighbourhood); deficient knowledge about the causes, diagnosis, treatment, and prevention of MiP and its risks for the fetus; the need to consult the hospital in symptomatic cases and at risk of obstetric complications; and a diagnosis involving out-of-pocket health expenses. These findings converge with other qualitative studies on malaria in Colombia, in which knowledge focused on mosquitoes, fever and weakness; but poor on other topics, such as risk groups, surveillance, and prevention [38].

The results also converge in the fact that community health actions to prevent and control malaria are not reported, and biomedicine is consulted only in advanced symptomatic cases, except for some people who have had previous malaria or who perceived a high risk of contracting malaria (those attend diagnosis and treatment services in a timelier manner) [38]. This situation has also been described in qualitative MiP approaches in other countries, where a low level of knowledge about MiP and a low perception of the risk of infection are highlighted [36, 37]. Together, these QUAN and QUAL findings demonstrate multiple challenges for health education, information, and communication programmes, which can identify thematic axes for their programs in the results of this research as well as the importance of adjusting these educational strategies to each reality or sociocultural context.

This interdependence of QUAN-QUAL results demonstrates the relevance of mixed studies in which feedback is presented on the strengths of each method, such as the statistical magnitude of the problem and its generalization possibilities, with the explanatory depth of qualitative analyses that emanate from the community and from daily lives. With this in mind, the main purposes of mixed studies are materialized, that is, understanding or explanation with greater breadth, depth and complexity; triangulation to identify convergences, correspondences or corroborate results; complementarity to improve or clarify results of one approach with those of the other; expansion of theoretical and explanatory frameworks, and practical usefulness for articulating diverse points of view and not segmenting the phenomenon of study or reducing it to the statistical or experiential field [28].

The above also applies to the articulation of the results of the Pender-Walker construct with the meanings of pregnancy and motherhood, as the meanings expand and allow us to explain some findings of the six dimensions of lifestyles; at the same time, the Pender-Walker construct facilitates the approach to topics that were not possible to address ethnographically.

In this sense, the Pender-Walker lifestyle scale was valid and reproducible in the pregnant women evaluated; this means that the six dimensions of the construct had an adequate psychometric structure and that the items were specific for each dimension (they measure what they intend to measure) in this population. In addition, very low scores were found for the dimension of physical activity; regular in nutrition, stress management, interpersonal relationships, and health responsibility; and high results were only found in the dimension of spiritual growth, which reflects the magnitude and severity of the unhealthy lifestyles of the study group.

These data are difficult to contrast with the scientific literature in this field because the lifestyles of pregnant women have been studied mainly on issues of hypertension, diabetes, obesity, anxiety, and depression or to evaluate the effectiveness of educational and nutritional interventions, without studies of MiP (as can be corroborated by performing a systematic search in PubMed with the following syntax: (life style[Title/Abstract]) AND (pregnant women[Title/Abstract])). Something similar occurs when restricting the search to the scale used in this research, which has not been used in similar populations. Despite this, the results reveal a worrying reality if they include a series of risky lifestyles for maternal health, which must be considered in antenatal care programmes.

The only dimensions of the Pender-Walker construct that showed a statistical association with MiP were stress management and health responsibility, with higher scores among pregnant women with malaria. This could be aided by the convergence of the following practices of some pregnant women: higher use of different health resources such as consulting doctors, better access to health information, attendance at health education workshops, and more frequent screening for malaria, among others related to diagnosis of MiP and the items of these two dimensions of lifestyles [34].

In the four remaining dimensions, no differences were found between women with and without MiP, indicating that the profiles of physical activity, nutrition, interpersonal relationships, and spiritual growth tended to be constant throughout the entire group evaluated. These unhealthy lifestyles may constitute a reflection of group processes that are related to sociocultural and economic aspects that influence nutritional limitations, low social support, and little focus on maternal health, among other aspects that require further study among MiP researchers.

Within the framework of SDH, it is important to highlight the absence of protective processes at the singular level, except for the positive meanings of pregnancy and motherhood. Although the matrix of SDH processes invites us to detect dialectical tensions between protective and destructive processes [1–3], in this population the protective are practically non-existent, which has several implications: (i) no mechanisms are identified to promote actions in favor of health from a popular initiative; (ii) it is not clear if these communities really exercise some important degree of autonomy regarding their health, it is not clear if the use of their autonomy is limited to the preference for traditional-popular medicine, or if this preference is more a consequence of the structural problems of the Colombian health system; (iii) apparently there are no real possibilities of fighting against the negative aspects of the social order that is reproduced from the general to the singular, generative-transformative actions are not identified from the singular level to the particular, there is no dialectical tension between production-reproduction of social processes related to the control of MiP.

In ethical and political terms, it is not possible to analyze lifestyles within the framework of individual autonomy because the control of MiP implies a high dependence of the actors of the health system, the husbands and relatives of pregnant women, and its social and political context, including some cultural and economic restrictions regarding the use of biomedicine. This is key to designing subsequent studies and analysing public health policy challenges for this type of disease.

## Limitations and strengths

The limitations included the exploratory nature of the statistical associations and the difficulty of including pregnant women who do not visit a local hospital for antenatal care in the QUAL component. Despite this, the main strengths of the research are the trends in the epidemiological profile and narratives of the daily lives of pregnant women, to illustrate other dimensions of the MiP problem that are not captured with traditional biomedical research and are not included in health planning.

It is important to highlight that the subjective and intersubjective dimensions are not captured by the traditional epidemiology approaches that predominate in MiP, whereas the objective dimension is excluded from social studies of the disease; aspects that were corrected using the mixed design. Furthermore, the epidemiological profile of MiP was articulated with the QUAN + QUAL approach to lifestyles, attempting to reflect the indissoluble nexus of the objective, subjective, and intersubjective dimensions (Disease Illness and Sickness) [1–4, 39].

## Conclusion

A high frequency of MiP was observed, and some groups with a higher occurrence of the disease were identified. This coincides with the presence of unhealthy lifestyles (according to the dimensions of the Pender-Walker construct and others specific to MiP), poor knowledge about malaria, low perception of the risk of getting sick, and meanings and experiences about MiP, maternity, and pregnancy that show a high clinical, cultural, and socioeconomic burden for the women studied. This epidemiological profile and the approach to lifestyles from the postulates of the SDH show that pregnant women exposed to malaria suffer serious social, cultural, and health injustices that are not possible to impact with the current model of malaria control in Colombia guided by aetiopathogenic, biomedical, positivist, and utilitarian theories. These injustices apply to all pregnant women in the studied area, as they all share the described structural problems: machismo, gender roles, conceptions of motherhood, and the evasion of responsibilities by health authorities (Ministry of Health, through the HPE, HSPI, and municipal health secretariats). Although some have a greater ability to overcome these issues due to their level of income, family support, or education level, they are a minority, given that MiP in Colombia is concentrated in poor, rural areas far from hospitals.

## Data Availability

All relevant data supporting the conclusions of this article are included within the article. Any additional information is available from the corresponding author upon reasonable request.

## Abbreviations

MiP: Malaria in pregnancy
SDH: Social Determination of Health
